# Central nervous system pericytes express soluble ST2 in inflammation and injury

**DOI:** 10.1186/s13041-026-01291-5

**Published:** 2026-03-19

**Authors:** Deidre Jansson, Blake Highet, Susan Li, Taylor J. Stevenson, Leon C. D. Smyth, Justin Rustenhoven, Sheryl Feng, Richard Daneman, Cayce Dorrier, Andrew Clarkson, Emma Scotter, Mike Dragunow

**Affiliations:** 1 Department of Pharmacology and Clinical Pharmacology, The University of Auckland, Auckland, New Zealand; 2 Centre for Brain Research, The University of Auckland, Auckland, New Zealand; 3 Department of Anatomy and Medical Imaging, The University of Auckland, Auckland, New Zealand; 4 Department of Pathology and Immunology, Washington University in St. Louis, St. Louis, USA; 5 Department of Psychiatry and Behavioral Science, University of Washington, Building 101, room 3W56 1660 South Columbian Way, Seattle, USA; 6 Department of Neurosciences, Department of Pharmacology, University of California, San Diego, USA; 7 Department of Anatomy, Brain Health Research Centre and Brain Research New Zealand,, University of Otago, Dunedin, New Zealand; 8 School of Biological Sciences, The University of Auckland, Auckland, New Zealand

**Keywords:** PDGF-BB, IL1RL1, IL-33, IL-4, TGF-alpha

## Abstract

Brain pericytes are mediators of neuroinflammation, as evidenced in vitro, in animal models and humans. We and others have identified the platelet-derived growth factor (PDGF)-BB -PDGF receptor beta (PDGFRB) pathway as a key modulator of inflammatory cues in human brain pericytes. We investigate the receptor for interkeukin-33 (IL-33), interkeukin-1 receptor-like 1 (IL1RL1; also known as ST2) as a highly upregulated transcript in response to PDGF-BB stimulation in pericyte cultures. We show that pericytes express transcripts for both the membrane bound form of the receptor (ST2L) and the soluble form (sST2) that acts as a decoy and blocks IL-33 signalling. Human brain pericytes secrete sST2 in response to PDGF-BB, but also to transforming growth factor alpha (TGF) alpha and interleukin-4 (IL-4), although they are unresponsive to IL-33 treatment. We also examine pericyte expression of both *IL1RL1* transcripts using RNAscope in two different in vivo models of neuroinflammation, experimental autoimmune encephalitis (EAE) and photothrombotic stroke. We present data showing that in rodents pericytes increase transcript expression predominantly for soluble *IL1RL1* in inflammatory and stroke models. Our results highlight a novel expression pattern of the soluble and membrane bound forms of the IL-33 receptor in vitro and in combination with our observations in vivo suggest that cerebrovascular pericytes may negatively regulate IL-33 signalling following injury or inflammatory insults to the brain.

**Supplementary Information:**

The online version contains supplementary material available at 10.1186/s13041-026-01291-5.

## Background

Pericytes are critical cellular components of the brain vasculature, being anatomically situated to communicate between the central nervous system and the periphery. However, pericytes also regulate the blood–brain barrier, cerebral blood flow, and contribute to amyloid clearance and neuroinflammatory processes [1, 2]. Platelet-derived growth factor receptor beta (PDGFRβ), a commonly used pericyte marker, is activated by endothelial-derived PDGF-BB during angiogenesis, and is required for proper vessel formation and pericyte vessel coverage during development [3–5]. Binding of PDGF-BB dimers to PDGFRβ/PDGFRα hetero- or homodimers initiates a cascade of signalling that includes phosphoinositide 3-kinase (PI3K), mitogen-activated protein kinase (MEK), protein kinase C (PKC) and Janus kinase (JAK)–signal transducer and activator of transcription (STAT) pathways to direct growth and survival, inflammation, proliferation, response to cell stress, and tumour suppression outcomes [6]. While genetic deletion of PDGF-BB or PDGFRβ is embryonic lethal, reduced PDGF-BB/PDGFRβ expression generates developmental phenotypes that include abnormal vessel formation, blood–brain barrier permeability, and neurodegeneration [7]. However evidence of PDGF-BB/PDGFRβ signalling in injury, inflammatory, and mitogenic responses as well as survival signals suggests a versatility that is not yet fully understood [8–11].

We have previously shown that in vitro, stimulation of human brain pericytes with PDGF-BB acutely induces both inflammatory and proliferation signals, and that knock-down of PDGFRβ blocks both of these processes [11, 12]. Although the importance of the PDGF/PDGFRβ signalling axis is clear, what is less understood is the role in homeostasis and response to injury or inflammation at the cerebrovascular milieu. For instance, a recent study in mice has suggested that PDGFRβ signalling is important for repair after ischemic injury, and could be a target for vascular healing [10]. However, PDGF-BB knock-down in adult mice resulted in reduced pericyte coverage and BBB permeability without effects on vascular function such as vessel dilation or calcifications and adult-induced knock-down of PDGFRβ in otherwise healthy mice displayed no measurable deficits [10, 13]. Only when exposed to an ischemic insult did the PDGF-BB/PDGFRβ deficient mice show larger infarct volumes and delayed recovery. To better understand the functional outputs of PDGF-BB/PDGFRβ signalling we inspected a previously obtained RNAseq dataset of pericytes treated with PDGF-BB [11]. One gene of interest that was upregulated in response to PDGF-BB treatment at 24 h in pericytes was interleukin-1 receptor-like 1 (*IL1RL1*, also known as *ST2*), which encodes a receptor for Interleukin-33 (IL-33). IL-33 is a member of the IL-1 cytokine family, and known to be involved in a variety of inflammatory processes, including T helper type 2 (Th2) immune responses, and clinically associated with inflammatory and injury related diseases such as stroke, multiple sclerosis, and Alzheimer’s disease [14–20].

ST2 is expressed as several different isoforms, two of which can be found in humans in the central nervous system. The membrane bound form ST2L is a type-1 transmembrane protein and a known receptor for IL-33 (transcriptional start site + 35)[21]. However soluble ST2 (sST2) that results from a separate promoter region [22] and lacks a transmembrane domain, is a secreted isoform which acts to sweep up extracellular IL-33, and blocks signalling through the membrane-bound receptor [23]. Typically, IL-33 would be released into the extracellular space during cell injury or necrosis where it can bind to heterodimers of ST2L and IL-1RAcP (IL-1 receptor accessory protein) at the membrane. This would initiate a cascade of cellular events including recruitment of adapter proteins (myeloid differentiation primary response 88 (MyD88), interleukin receptor-associated kinase) IRAK1/4, TNF-receptor-associated factor 6 (TRAF6)) and downstream transcription factors extracellular signal-related kinases (ERKs), c-jun N-terminal kinase (JNK), nuclear factor kappa light chain enhancer of activated B cells (NFκB), and p38 mitogen-activated protein kinases (p38 MAPK))[24].

In cases of experimental autoimmune encephalomyelitis (EAE), blocking IL-33 release during induction of EAE results in a milder disease phenotype with reduced inflammation and slowed demyelination, while IL-33 treatment worsens these outcomes [25]. In contrast, IL-33 treatment of post-ischemic mice was generally neuroprotective, promoted Th2 immune responses and reduced pro-inflammatory cytokines [26]. However, deletion of ST2 specifically in CNS cells results in larger infarct volumes, indicating that ST2 mitigates post-ischemic injury responses [27]. Meanwhile higher serum sST2 is predictive of worse outcomes following stroke in humans [28, 29] and in vascular injury in mouse models, together suggesting that blocking IL-33 signalling either through deletion of the receptor, or through actively sweeping up extracellular IL-33 with sST2 impairs stroke recovery.

Unfortunately, most studies to date regarding ST2 expression do not discern between the two isoforms, despite their opposing effects on IL-33 signalling. In addition, while there is evidence of IL-33 expression in the CNS in endothelia, astrocytes and microglia, and ST2 expression in astrocytes, microglia and even oligodendrocytes [30, 31], there is currently no evidence of ST2 expression in vascular pericytes. We therefore characterized both ST2L and sST2 expression and secretion in pericytes in in vitro and in vivo conditions that have documented PDGF-BB and IL-33 expression. Our study shows that both ST2L and sST2 are strongly induced in response to PDGF-BB, and other inflammatory mediators (transforming growth factor alpha (TGFα, interleukin-4 IL-4)) in pericytes in vitro, and in spinal cord vascular PDGFRβ expressing cells following EAE. We also show that both *sST2* and *ST2L* transcripts are induced following photothrombotic stroke, however, this response was not specific to PDGFRβ positive vascular cells and found mostly in contralateral regions.

## Results

### Induction of Interleukin-1 receptor-like 1 by PDGF-BB

**Fig. 1:**
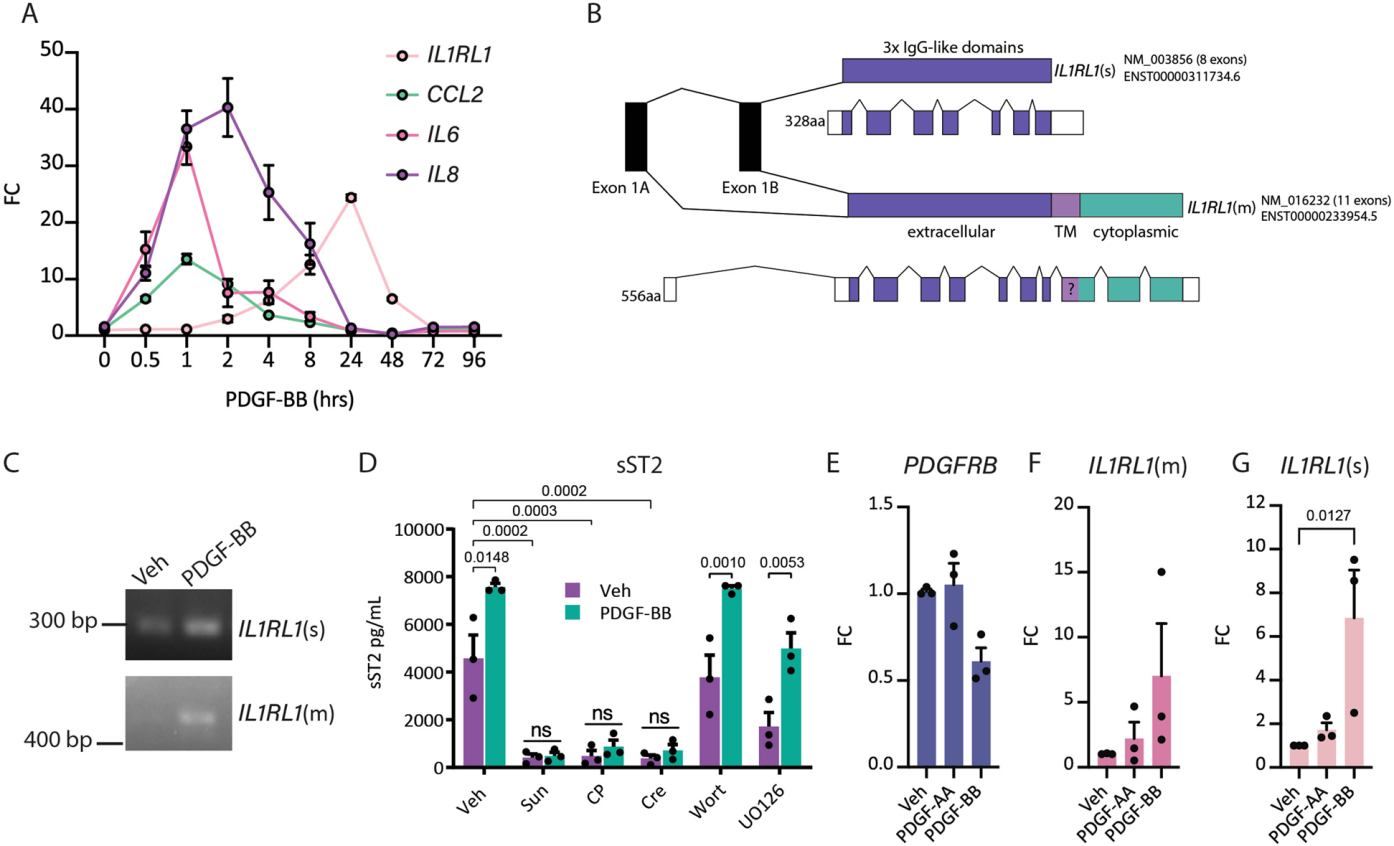
Secreted ST2 (sST2) is the main isoform of *IL1RL1* induced by PDGF-BB in pericytes in vitro.**A** Time-course of PDGF-BB (10 ng/mL) treatment in pericytes with RT-qPCR detecting *CCL2*, *IL6*, *IL8*, and *IL1RL1* (all isoforms). Significance is indicated in the following:IL1RL1: 4 (*p* = 0.0323), 24 (*P*=0120), 48 h (*p *= 0.0277); CCL2: 0.5 (*P*=0.0074), 1 (*p *= 0.0049), 8 (*P*=.0019), 48 h (*p* = 0.0077)2 (*p*= 0.0004), 4 h (*P*=0.0007); IL6: 0.5 (*p* = 0.0410), 48 h (*p* = 0.0176)1 h (*P*= 0.0086); IL8: 0.5 (*P*= 0.0119), 2 (*p* = 0.0155), 4 h (*p*= 0.0347), 1 h (*P* = 0.0085)** B**)Schematic of ST2 isoforms: IL1RL1(s) and IL1RL1(m) demonstrating different transcriptional start sites and exon (coloured blocks)-intron(lines) regions. Exon1A being the start site for *IL1RL1*(m) and exon1B being the start site for *IL1RL1*(s).** C**) Standard PCR to amplify specific regions of the *IL1RL1* mRNA at 35 cycles to verify specificity of primer regions for either *IL1RL1*(m) *or IL1RL1*(s).** D**) ELISA of sST2 in pericyte conditioned media pre-treated with inhibitors (Sun (Sunitinib) (100 nM), CP-673451 (CP, 100 nM), crenolanib (Cre, 100 nM), Wortmannin (Wort, 100 nM), UO126 (10 μM)) for 1 h, then PDGF-BB for 24 h. G-I) RT-qPCR analysis of** E**) *PDGFRB*,** F**) *IL1RL1*(m) and** G**) *IL1RL1*(s) in pericytes treated with either vehicle, PDGF-AA, or PDGF-BB (10 ng/mL for 24 h), FC = fold change over vehicle (n = 3 cases for all experiments. Statistical tests: A) Two-way ANOVA with uncorrected Fisher’s LSD, D) Two-way ANOVA with Dunnett’s multiple comparisons test E–G) ANOVA with Kruskal–Wallis correction for multiple comparisons

*IL1RL1* or its translated protein, ST2, is a known receptor for IL-33, and to our knowledge, *IL1RL1* expression and its role in brain pericyte biology was unknown. We examined gene expression of typical inflammatory signals from 0–96 h of PDGF-BB treatment to discern a timeline of *IL1RL1* induction. Transcript levels of *CCL2*, *IL-6, IL-8* and *IL1RL1* indicate time-dependent gene regulation (Fig. 1A). This is consistent with previous work in pericytes showing inflammatory responses with PDGF-BB treatment [11, 32]. *IL1RL1* demonstrated delayed induction, with gene expression plateauing at 24 h. Interestingly we did not see an induction of *IL33* in brain pericytes, in the RNAseq data, which had been shown by other groups [33].

*IL1RL1* encodes two protein isoforms from a shared mRNA sequence, including the membrane-bound IL33 receptor (ST2L), and a shorter, soluble, decoy isoform (sST2), which arise due to a dual promoter system and alternative splicing [34] (Fig. 1B). The transcripts isoforms will be referred to as *IL1RL1*(s) (soluble) and *IL1RL1*(m) (membrane) for clarity for the remainder of the manuscript. Using isoform-specific primers, we discovered that although both isoforms were expressed in primary human brain pericytes in response to PDGF-BB treatment, basal levels of *IL1RL1*(m) were undetectable, while the soluble form (*IL1RL1*(s)) was more highly expressed in both basal and treated conditions (Fig. 1C, Supplementary Fig. 1). Using RT-qPCR we saw that PDGF-BB significantly increased transcript levels of *IL1RL1*(s), but not *IL1RL1*(m) (Supplementary Fig. 2). At the protein level, we detected sST2 secreted by pericytes into the media under vehicle treated conditions, that was increased in response to PDGF-BB treatment (Fig. 1D, Supplementary Fig. 2). Pharmacological inhibition of PDGFRβ with sunitinib, crenolanib or CP-673451 however completely abolished sST2 secretion, while inhibition of the MAPK/ERK or PI3K pathway had no effect (Fig. 1D). Since PDGF-BB is not specific to PDGFRβ but can bind both PDGFRβ/PDGFRα heterodimers and PDGFRα homodimers, whereas PDGF-AA binds specifically PDGFRα [35], we postulated that perhaps sST2 could be induced by PDGF-AA as well. We therefore treated pericytes with PDGF-BB or PDGF-AA and measured gene expression of *IL1RL1*(s) and *IL1RL1*(m). Although we observed that PDGF-AA moderately induced *IL1RL1*(s) (mean increase 1.7-fold over vehicle, p = 0.34) and *IL1RL1*(m) expression (mean increase 2.2 over vehicle, p = 0.15) this did not reach statistical significance (Fig. 1E-G). Together our data suggests that PDGF-BB treatment of pericytes results in increases in soluble ST2 through PDGFRβ.

### sST2 expression in pericytes

**Fig. 2:**
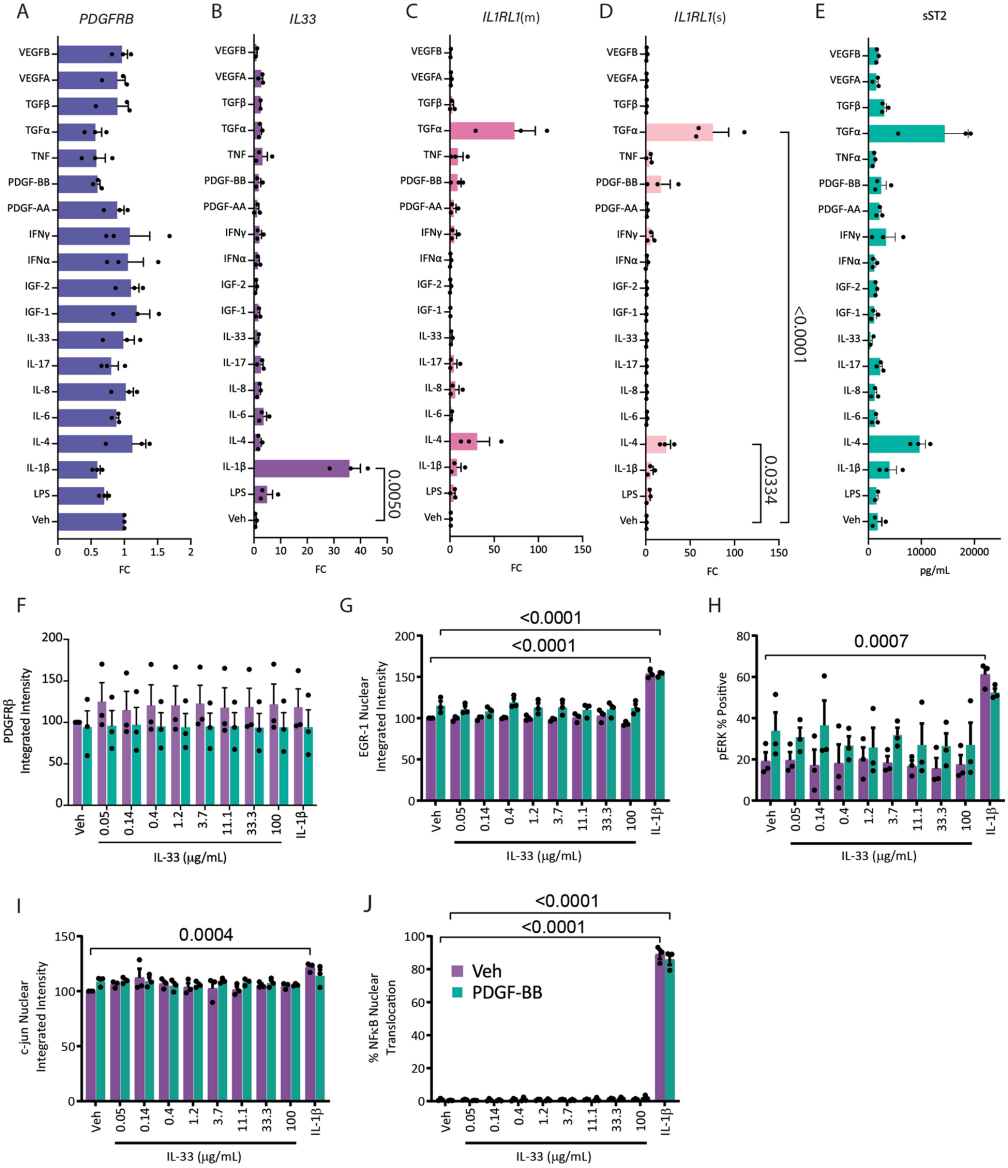
Pericytes secrete sST2 but do not respond to IL-33 in vitro.** A-D**) Pericytes were treated with a cytokine screen to detect changes in gene expression of** A**) *PDGFRB*,** B**) IL33,** C**) *IL1RL1*(m) and** D**) *IL1RL1*(s) after 24 h.** E**) ELISA screen for sST2 in pericyte conditioned media after 24 h of treatment.** F**) Pericytes were treated with PDGF-BB or vehicle for 24 h, then IL-33 or IL-1β to measure downstream activity of** G**) EGR-1,** H**) ERK,** I**) c-jun, or** J**) NFκB (n = 3 cases, statistical tests:** A-E**) ANOVA with Kruskall-Wallis for multiple comparisons,** F-J**) Two-way ANOVA with Dunnett’s correction for multiple comparisons)

Although the transmembrane form of ST2 is a receptor for IL-33, and is generally observed to elicit T helper type 2 lymphocyte responses [14], expression has also been reported in innate and adaptive immune cells such as mast cells, macrophages, microglia, dendritic cells, eosinophils, natural killer T cells, basophils, as well as endothelial cells in response to a range of stimuli [27, 36, 37]. However, whether pericytes respond to the cytokine IL-33, or other stimuli by secreting sST2 has never been tested. To further investigate the regulation of *IL1RL1* expression in pericytes we screened 18 cytokines and growth factors including PDGF-BB and IL-33 for their effects on *IL1RL1*(s) and *IL1RL1*(m) gene expression and sST2 secretion (Fig. 2A-E). The *IL33* transcript was significantly induced by IL-1β (Fig. 2B). Alternatively, IL-33 treatment did not result in *IL1RL1* transcript increases. Expression of both *IL1RL1*(s) and *IL1RL1*(m) was increased in response to transforming growth factor alpha (TGFα(*IL1RL1*(m) p = 0.06, *IL1RL1*(s) *p* < 0.0001) and to a lesser extent IL-4 (*IL1RL1*(m) *p* = 0.14, *IL1RL1*(s) *p* = 0.03) (Fig. 2C-D). Secretion of sST2 while not reaching statistical significance after correction for multiple comparisons, followed the same pattern as the transcript data (Fig. 2E) being increased in response to IL-4 and TGFα.

To test whether brain pericytes responded to IL-33 treatment at all we tested known downstream effectors of IL-33 signalling after treatment with IL-33. Whilst pericytes express both the transcript isoforms for the membrane bound and secreted form of *IL1RL1*, there was no response to IL-33 treatment in terms of either PDGFRβ expression, or activity of downstream effectors EGR-1, ERK, c-jun, or NFκB, activity (Fig. 2F-J) after 1 h treatment. This is surprising since pericytes do express at least the transcript for *IL1RL1*(m) and the co-receptor *ILRAP* at the mRNA level (according to RNAseq data [11]), and reports in a recent study of lung pericytes responding to IL-33 treatment [33]. Whether human brain pericytes respond to IL-33 treatment autonomously has not been tested. We therefore performed a time course of IL-33 treatment to match the experimental paradigm previously reported [33] in our brain pericyte cultures and found no response to IL-33 in terms of pAKT or pERK as early as 5 min to 60 min of treatment (Supplementary Fig. 3). We then hypothesized that since baseline ST2L membrane expression was undetectable in pericytes, perhaps stimulation was required to generate ST2L receptor protein to elicit an IL-33 response through its receptor. We therefore treated pericytes with either vehicle, TGFα, IL-4 or PDGF-BB for 24 h, then tested for response to IL-33. There was no response to IL-33 in any of the conditions by measures of NFκB nuclear translocation, phosphorylated ERK, or Akt (Supplementary Fig. 4). This data suggests that pericytes may be upregulating expression of sST2 to block IL-33 signalling in surrounding, non-pericyte cells.

### Pericytes express ST2 in response to neuroinflammation and injury

**Fig. 3:**
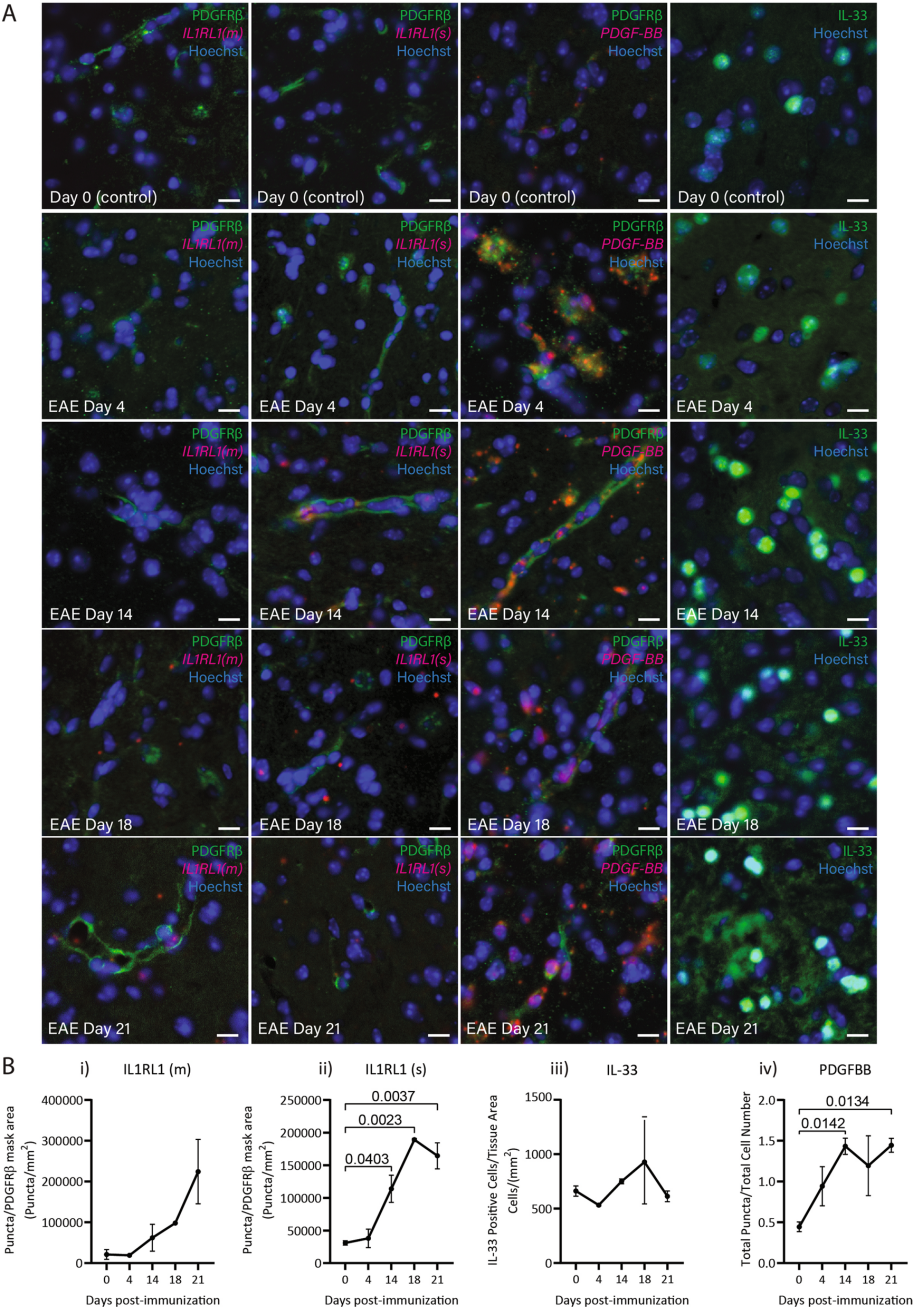
*IL1RL1* mRNA expression follows *PDGFB* in EAE.** A**) Representative images of mouse spinal cord labelled with PDGFRβ or IL-33 (green) and RNAscope probes for either *IL1RL1*(m), *IL1RL1*(s), or *PDGFB* (red), overlaid with Hoechst for nuclei (blue).** B**) Quantification of images in 3A), for RNAscope puncta for i) *IL1RL1*(m) and ii) *IL1RL1*(s) within the PDGFRβ-positive pericytes after disease induction, and iii) quantification of IL-33 positive nuclei, or iv) *PDGFB* total RNAscope puncta. Statistical analysis: ANOVA with Dunnett’s correction for multiple comparisons (n = 2–3 mice per timepoint, 2 sections per mouse)

Pericytes respond to different types of stimulation including PDGF-BB, TGFα, and IL4 exposure by releasing soluble ST2 in vitro. To determine whether this occurs in vivo we examined both *IL1RL1*(m) and *IL1RL1*(s) RNA expression in an animal model of multiple sclerosis, experimental autoimmune encephalitis (EAE), in which we would expect inflammatory responses including elevated levels of cytokines in the CNS such as IL-33, and in which PDGF/PDGFRβ signalling is involved [38–40]. We examined EAE spinal cord tissue prior to (Day 0) and 4, 7, 14, 21 days post immunization using RNAscope to identify specific isoforms of *IL1RL1* (with positive and negative controls shown in Supplementary Fig. 5) coupled with immunohistochemistry for PDGFRβ and lectin to label pericytes and vasculature, respectively (Fig. 3A). In EAE there was a rapid increase in both *IL1RL1*(m) and *IL1RL1*(s) RNA from day 4 to 21, although like the in vitro conditions the secreted form was in higher abundance than the membrane form (Fig. 3Bi-ii). The increase in *IL1RL1*(s) was preceded by an increase in *PDGFB* RNA, although there was no significant change in IL-33 protein expression (Fig. 3Biii-iv). We also confirmed expression of each *IL1RL1* isoform in pericytes with an additional pericyte marker, CD13 and confocal imaging (Supplemental Figs. 6 and 7) While we did detect RNAscope signal in the vasculature by co-labelling for PDGFRβ and CD13, there was also expression outside the vessels, likely in other cell types not examined here.

While IL-33 signalling plays a role in chronic inflammatory responses, there is also evidence of IL-33 pathway activation in response to injury such as ischemia or stroke [26, 41]. Studies to date have examined IL-33 and ST2L expression in microglia, astrocytes and immune cells, but to our knowledge, vessel-associated ST2 has not been examined. We therefore employed a photothrombotic stroke model to test whether soluble ST2 is induced in response to acute ischemic insult. Mice aged 10–12 weeks were subjected to photothrombotic stroke and allowed to recover for 1, 3, 5, or 7 days, followed by RNAscope for *IL1RL1*(s), *IL1RL1*(m) and *PDGFB* and staining for lectin and PDGFRβ (n = 5 per timepoint). We observed co-localisation of RNAscope probes within CD13-positive pericytes (Supplementary Figs. 8 and 9), however, due to the extent of the injury and the autofluorescence of red blood cells in the stroke region, quantification was not possible. Representative images from sham, and days 1, 3, 5 and 7 post-stroke are presented in supplementary data to demonstrate the extent of the injury and the infarct region (Supplementary Fig. 10).

**Fig. 4:**
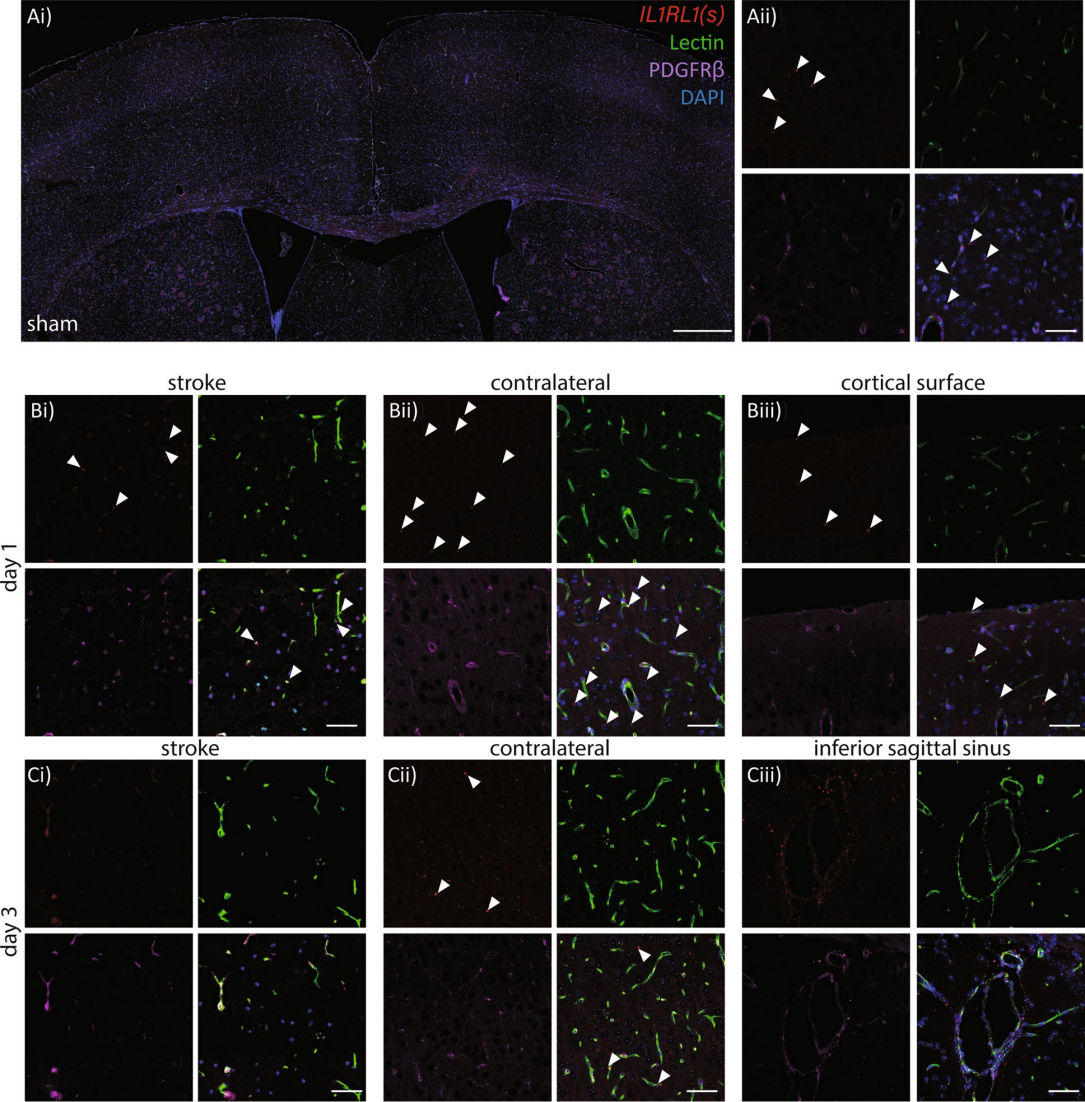
*IL1RL1*(s) is expressed in the brain vasculature and neuropil acutely following stroke.** A**) Sham expression of *IL1RL1*(s) co-labelled with lectin (green), PDGFRβ (purple) and RNAscope probe (red), Ai) low magnification of dorsal cortical region, scale bar = 500 μm, Aii) higher magnification to visualize the probe, scale bar = 50 μm.** B**) Day 1 post-stroke *IL1RL1*(s) probe colabelled as in (A) in i) stroke region, ii) contralateral cortical, and iii) contralateral cortical surface regions, scale bar = 50 μm.** C**)Day 3 post-photothrombotic stroke *IL1RL1*(s) probe colabelled as in (A) in i) stroke region, ii) contralateral cortical region, and iii) inferior sagittal sinus, scale bar = 50 μm

Qualitative assessment of *IL1RL1*(s) probes revealed little to no probe detection in the sham tissue (Fig. 4A i-ii), however day 1 post-stroke there was probe detection in the contralateral cortical regions and meninges (Fig. 4B i-iii). While some probe appeared associated with vessels by lectin or PDGFRβ stain, much of the probe was detected outside vessel areas. By day 3 post-stroke *IL1RL1*(s) probe was undetectable in the ipsilateral side, and appeared present but to a lesser extent in the contralateral side (Fig. 4C i-iii). Of note, we detected considerable *IL1RL1*(s) probe surrounding the inferior sagittal sinus and associated large vessels. By day 5, little to no probe could be detected in either the ipsilateral or contralateral regions and this was similar at day 7.

**Fig. 5:**
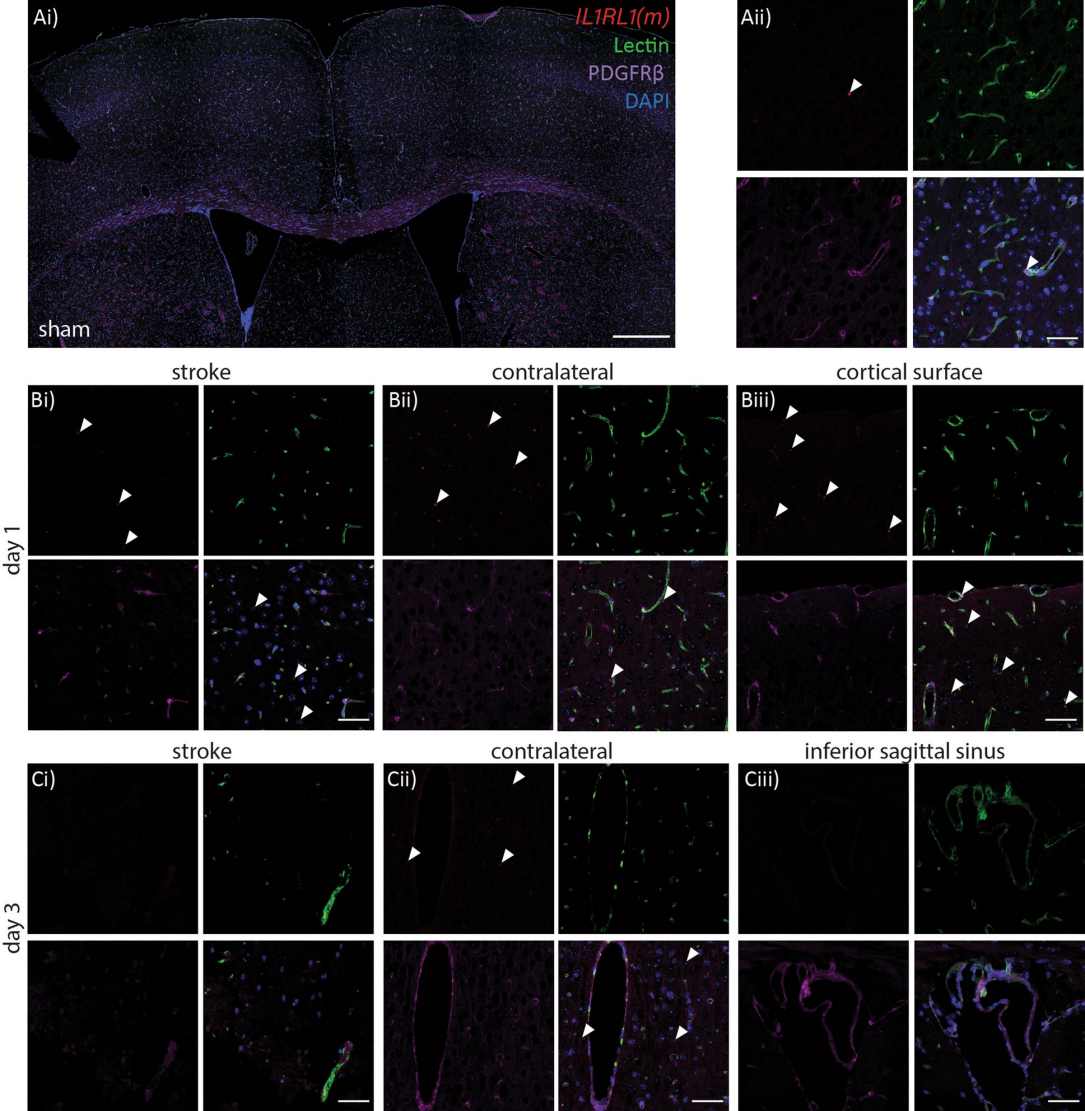
Membrane-bound *IL1RL1* mRNA in situ following stroke** A**) Expression of *IL1RL1*(m) mRNA in sham tissue colabelled with Lectin (green), PDGFRβ (purple) and RNAscope probe (red), Ai) low magnification of dorsal cortical region, scale bar = 500 μm, Aii) higher magnification to visualize the probe, scale bar = 50 μm.** B**) Day 1 post-stroke *IL1RL1*(m) probe colabelled as in (A) in i) stroke region, ii) contralateral cortical, and iii) contralateral cortical surface regions, scale bar = 50 μm.** C**) Day 3 post-photothrombotic stroke *IL1RL1*(m) probe co-labelled as in (A) in i) stroke region, ii) contralateral cortical region, and iii) inferior sagittal sinus, scale bar = 50 μm

Examination of *IL1RL1*(m) in sham tissue was similar to *IL1RL1*(s) in that little to no probe could be detected in any brain regions (Fig. 5A i-ii). At day 1 post-stroke, we saw some detectable probe in the stroke region, with a similar induction to *IL1RL1*(s) in the contralateral side and meninges (Fig. 5B i-iii). By day 3 *IL1RL1*(m) probe detection was sharply reduced and by day 5 was similar to sham detection levels (Fig. 5C i-iii). In contrast to *IL1RL1*(s), no *IL1RL1*(m) probe could be detected in the inferior sagittal sinus or associated vasculature.

**Fig. 6:**
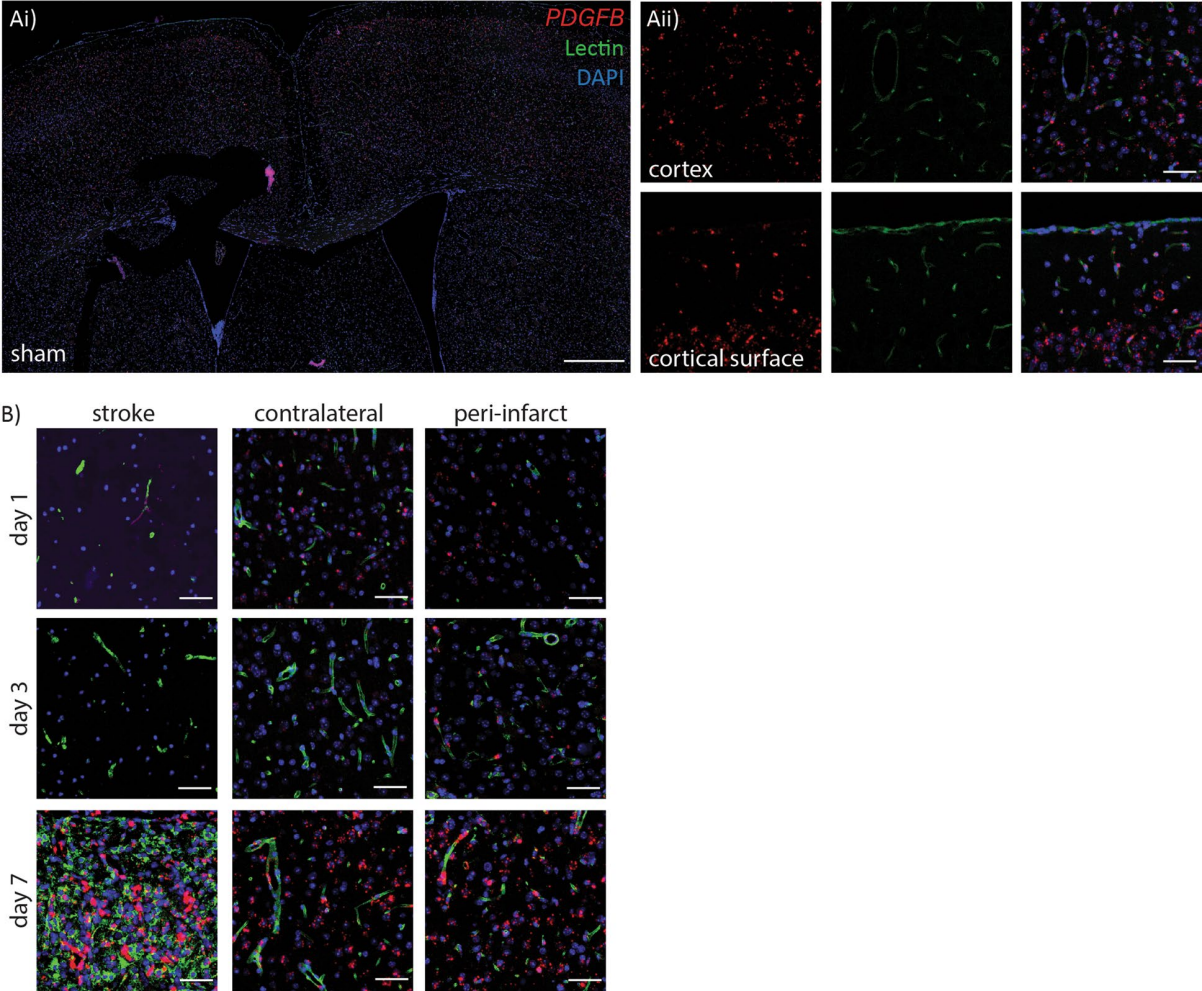
*PDGFB* mRNA expression following stroke** A**) Representative images of expression of *PDGFB* mRNA in sham tissue co-labelled with lectin (green) and RNAscope probe (red) throughout the brain parenchyma, scale bar = 500 μm, Ai) lower magnification of the dorsal region of the cortex, and Aii) higher magnification image to visualize the probe, scale bar = 50 μm.** B**)*PDGFB* probe in stroke region co-labelled with lectin (green) and RNAscope probe (red), peri-infarct areas and contralateral cortical regions at day 1, 3 and 5 post-ischemia, scale bar = 50 μm

*PDGFB* probe detection was quite different to that of *IL1RL1*(m) and *IL1RL1*(s). In sham tissue *PDGFB* probe was abundant in all brain regions, associated with vessels and in the neuropil, with reduced density close to the cortical surface (Fig. 6A i-ii). At day 1 post-stroke no *PDGFB* could be detected in the ipsilateral hemisphere within the stroke region but could be detected at similar density to sham in the peri-infarct area, and in the contralateral cortical regions (Fig. 6B). By day 3 post-stroke no *PDGFB* probe could be detected in the stroke region and appeared reduced in the peri-infarct and contralateral cortical areas. By day 5 post-stroke *PDGFB* levels reached a similar density as sham in all areas except for the stroke region which remained undetectable. At day 7 post-stroke there was a substantial increase in *PDGFB* probe signal in the stroke region specifically with contralateral and peri-infarct levels remaining similar to day 5 and sham conditions (Fig. 6B).

## Discussion

There is growing evidence that pericytes are functionally diverse, critical for formation and maintenance of blood vessels, and physiologically reactive to maintain homeostasis, but also in response to injury and inflammation. Our current study builds on previous work of the transcriptional landscape of pericytes in response to a native ligand. We honed in on a novel pathway not previously identified in brain vascular cells and focused on conditions of disease and injury when these systems may go awry.

Our initial exploratory experiments confirmed a previously reported induction of the IL-33 receptor, *IL1RL1*(m), and the soluble secreted form *IL1RL1*(s) in response to PDGF-BB treatment of pericytes in vitro. The late induction of *IL1RL1*, peaking at 24 h, in contrast to other cytokines (*CCL2*, *IL6*) could suggest that *IL1RL1* gene expression is a secondary result of PDGF-BB treatment, for example being increased in response to PDGF-BB-induced inflammatory cytokines (TNFα, IL-1α, IL-1β IL-4 and IL-6) as has been previously reported in fibroblasts, epithelial and endothelial cells, and macrophages [42–44]. The difference in both basal and induced expression of *IL1RL1*(m) compared to *IL1RL1*(s) was consistent with the nature of the proteins themselves (membrane bound versus secreted), and previous work in other cell types: ST2L has generally lower basal expression and sST2 is more highly induced with cytokine treatment [45].

While we initially investigated *IL1RL1* because of a previously published dataset of PDGF-BB-induced genes in human brain pericytes, we also saw that *IL1RL1* gene expression induced in response to TGFα and IL-4. TGFα is an epidermal growth factor receptor (EGFR) ligand, and responsible for actions similar to both EGF and PDGF signalling pathways such as cell proliferation and differentiation and as such has been implicated in many cancers [46, 47]. In the brain TGFα expression has been observed in both developing and adult rat and human brain sections, in astrocytes and neurons predominantly, and can be detected in human cerebrospinal fluid [48, 49]. Administration of TGFα following ischemia–reperfusion injury reduced infarct size and memory impairment, increased angiogenesis and neurogenesis [50, 51]. However, circulating sST2 was associated with increased risk of stroke and administration of IL-33 can improve stroke outcomes [29, 52]. Thus, the beneficial effects of TGFα may not be through induction of soluble ST2 but through the ST2 receptor itself, and perhaps not through vascular cells. Overall, there seems to be an underappreciated role for TGFα in neuro-inflammatory pathways. In terms of ST2 induction, these authors could find no evidence of TGFα working through the PDGFRβ, although there is evidence of EGF-EGFR activation to stimulate ST2 expression (53).

The other cytokine that significantly induced *IL1RL1*(s) expression in our study was IL-4. While evidence of an involvement of IL-4 signalling in pericytes is limited, and the induction of sST2 even more so, one recent study demonstrated that IL-4 treatment in a model of intracerebral haemorrhage improved outcomes in a ST2-dependent manner [54]. This study failed to delineate between the membrane and soluble forms of ST2 however and did not interrogate vascular cells. Therefore, it remains to be seen whether IL-4 is specific to either membrane or soluble induction of ST2 in vivo, and if this occurs in pericytes.

In examining IL-33 signalling components we did not detect a response to IL-33 in human brain pericytes in vitro, nor did we detect IL-33 induction by PDGF-BB treatment in pericytes. RNAseq data from our group [11] showed that the co-receptor for IL-33, *ILRAP* was expressed at the mRNA level in pericytes in vitro, indicating that brain pericytes at least have the transcriptional components necessary to respond to IL-33. However, we have no evidence that IL-33 signalling occurs in these cells in vitro. The lack of IL-33 gene expression with PDGF-BB treatment was unexpected in light of a study showing IL-33 to be the most highly upregulated gene in response to PDGF-BB (5 days) in mouse lung pericytes (33), but consistent with our previous RNAseq study where no detectable IL-33 expression was detected at either 1 or 24 h after PFGF-BB treatment. These results highlight the potential differences in peripheral versus vascular cells as well as potential species differences and hence the different signalling properties.

We also examined two different in vivo models in which IL-33 signalling has been shown to play a role to determine whether pericyte ST2 (soluble and membrane isoforms) expression may be involved. Specifically, we chose the EAE model and the stroke model since blockade of IL-33 signalling has opposing effects in terms of improving health outcomes. In our EAE model, the increase in *IL1RL1*(s) may be acting as a negative feedback response to reduce or stop IL-33 signalling in either pericytes or surrounding immune populations, as blocking IL-33 with antibodies ameliorates disease outcomes in this model [55]. IL-33 expression is highest in the mouse CNS and spinal cord [14], and remain relatively constant in EAE by immunohistochemical measures, while ST2 is significantly elevated [55]. We did not detect a significant difference in IL-33 staining in the spinal cord sections after EAE induction up to 21 days, which is consistent with published results. In human studies however, elevated IL-33 can be detected in MS patient plasma, cerebrospinal fluid and brain tissue [18]. This discrepancy may be due to the difficulty in detecting released IL-33 in tissue sections, as opposed to ELISA-based techniques which do detect IL-33 in the spinal cord [56]. Previous work quantifying ST2 expression in EAE models conclude that ST2 is solely expressed in neurons (using immunofluorescence [55]) or astrocytes (measuring gene expression in culture [57]). Our data shows that both *IL1RL1*(m) and *IL1RL1*(s) mRNA also increase in pericytes. The inability to detect protein expression in the aforementioned studies may simply be due to the transient nature of secreted proteins and the difficulties of detection in tissue, but also due to the lack of interest historically in vascular cells. While we see both the membrane and secreted forms gradually increasing over time in the EAE model, the secreted form was more strongly induced out of the two. Taken together our data suggests that both membrane and soluble forms of ST2 may be playing a role in EAE progression, and targeting one over the other in future studies will help to tease apart disease mechanisms.

Our observations of *IL1RL1*(m) and *IL1RL1*(s) mRNA expression in the mouse model of stroke were less conclusive. Due to the severity of the injury, and autofluorescence of red blood cells in the stroke region, we could not quantify probe in pericytes While we observed *IL1RL1*(m) and *IL1RL1*(s) probe binding following injury in the stroke model, we could visualize limited pericyte-specific probe in this particular model. This could indicate that pericytes play a very minor role in IL-33 signalling in response to stroke, at least at the acute timepoints of up to 7 days.

Indeed in response to an ischemic insult, spinal cord injury, or TBI several studies indicate that IL-33 expression directly following the injury is protective [56, 58–60] [61], and there perhaps would not be a need for soluble ST2 to ‘turn off’ IL-33 signalling unless it continued to more chronic timepoints as is seen in MS. Our in vitro evidence indicating that mitotic and potentially anti-inflammatory molecules PDGF-BB, TGFα and IL4 can stimulate *IL1RL1* in pericytes suggests that this may be a compensatory mechanism of cells that have been exposed to chronic inflammatory conditions.

As for the differential effects of IL-33/ST2 signalling in the model of autoimmune dysfunction/chronic inflammation versus injury such as stroke, our data demonstrates a difference in the distribution of *IL1RL1* transcript in the two models. When present, *IL1RL1* RNA was detected throughout the spinal cord, consistent with chronic inflammation being more widespread. Comparatively, following stroke, *IL1RL1* RNA was limited to barrier regions and peri-infarct areas within the cortex. This points to a more regenerative function for IL-33 in the stroke model, as in models of cardiac injury where IL-33 reduced atherosclerosis and reduced glial scarring after ischemic injury [41, 59].

In conclusion, here we show that pericytes can express both membrane bound ST2L and secreted sST2 mRNA in response to a variety of inflammatory stimuli in vitro, and also under neuroinflammatory conditions in vivo. Whether expression of ST2 by cerebrovascular pericytes is advantageous is yet to be determined, and very few (if any) mechanistic studies have been carried out in human cell/tissue [45]. We suggest further studies where pericyte-specific soluble ST2 is targeted to define the role of pericytes in IL-33-mediated signalling homeostatic conditions as well as in response to inflammation or injury.

## Methods

Experiments conducted on human tissue in this study were approved by the Northern Regional Ethics Committee of New Zealand and carried out in accordance with approved guidelines. Tissue was derived from surgery for adult drug-refractive epilepsy and obtained with written informed consent from all patients and/or legal guardians.

### Human brain-derived pericyte culture

Brain tissue from the middle temporal gyrus (MTG), obtained following epilepsy surgery, was processed for the isolation and culture of brain pericytes, as previously described [62]. Briefly, after removal of visible meninges, approximately 2 g of tissue was dissociated mechanically (with two scalpels) and enzymatically (with 10 U/mL DNase I (Invitrogen), 2.5 mL papain (Worthington) in Hibernate A medium (Gibco) in a total of 10 mL). After passing through a 100 μm nylon strainer (Bector Dickinson) cells were incubated in complete DMEM (Dulbecco’s modified Eagle’s media with F12 supplement (DMEM:F12) with 10% fetal bovine serum (FBS)(Gibco), 1% Penicillin/streptomycin, L-glutamine 0.29 mg/mL (PSG) (Gibco)) and incubated at 37 °C with 5% CO_2_ to generate mixed glial cultures. Pericyte cultures were obtained after 4 passages as microglia and astrocytes do not proliferate and are no longer detectable. Experiments performed on pericytes were from passages 4–9, once microglia and astrocytes were depleted [63]. The purity and phenotypic characteristics of these cultures have been extensively characterised and validated in prior studies[11, 12, 64–69]. Cells were seeded into 96-well plates at 5000 cells/well in complete DMEM.

### Pharmacological inhibition of PDGFRβ

PDGFRβ inhibitors (Sunitinib (Sun), CP-673451 (CP), Crenolanib (Cre)), MAPK inhibitor (UO126), or PI3K inhibitor (Wortmannin) (Sigma) were used at 100nM final concentration. Cells were pre-treated with inhibitors for 1 h, then stimulated with PDGF-BB at 10 ng/mL for 24 h. Media was collected for ELISA and stored at is −20 °C.

### siRNA transfection

Synthetic ST2 siRNA (Santa Cruz, sc-40035) or a control siRNA (Santa Cruz, sc-37007) (50 nM) was incubated with Lipofectamine® RNAiMAX (0.03% final) (Life Technologies, CA, USA) in DMEM/F12 for 20 min at room temperature. siRNA was added to cells for 96 h prior to cell treatments, with a complete media change at 48 and 96 h.

### RNAseq

RNAseq data was obtained from post-mortem human brain pericytes treated for 24 h with PDGF-BB (10 ng/mL) [11], publicly available from the Gene Expression Omnibus (GES189712).

### PCR and RT-qPCR

RNA was extracted from pericytes using a RNeasy kit (Qiagen, 74004) following the manufacturer’s instructions. RNA was quantified using Nanodrop. cDNA was made from 2–3 µg DNase-1 (Promega, M6101)-treated RNA using the Superscript IV first strand synthesis kits (Invitrogen, 18090010). PCR was performed using the following primers (*IL1RL1(s)* fw GAAAAAACGCAAACCTAACT, *IL1RL1(s)* rv TCAGAAACACTCCTTACTTG, *IL1RL1(m)* fw AGGCTTTTCTCTGTTTCCAGTAATCGG, *IL1RL1(m)* rv GGCCTCAATCCAGAACATTTTTAGGATGATAAC) and Phusion DNA polymerase (New England Biolabs) according to manufacturer’s instructions, with the following cycle parameters: 98°C-30 s, (98°C-5 s, 60°C-20 s, 72°C-8 s (repeat 25-35x)), 72°C-8 min. Samples were visualized on a 1.5% agarose gel with RedSafe (VWR) and 1kb ladder (Invitrogen). Quantitative reverse transcriptase polymerase chain reaction (RT-qPCR) was performed as described previously [70].

For RT-PCR, primers are listed in Table 1.

**Table 1:** Human primers used in this study. Abbreviations, H, human; m, mouse; fw, forward; rv, reverse

Accession Number	Gene		Sequence (5’ to 3’)	Amplicon Length
NM_002982.3	*CCL2* (h)	Fw	CAGCCAGATGCAATCAATGCC	90 bp
		Rv	TGGAATCCTGAACCCACTTCT	
NM_002046.4	*GAPDH* (h)	Fw	CATGAGAAGTATGACAACAGCCT	113 bp
		Rv	AGTCCTTCCACGATACCAAAGT	
NM_000600.3	*IL6* (h)	Fw	GGCTGCAGGACATGACAACT	102 bp
		Rv	ATCTGAGGTGCCCATGCTAC	
NM_000584.3	*IL8* (h)	Fw	CAGAGACAGCAGAGCACACA	70 bp
		Rv	GTGAGATGGTTCCTTCCGGT	
NM_033439.4	*IL33* (h)	Fw	GAGAGAAACCACCAAAAGGCC	78 bp
		Rv	TGTTGACAGGCAGCGAGTAC	
NM_016232.5 and NM_003856.4	*IL1RL1* (h)	Fw	TGAGGACGCAGGTGATTACA	100 bp
		Rv	TTGCTCATCCTTGACCGTG	
NM_016232.5	*IL1RL1*(m) (h)	Fw	TCTGGATTGAGGCCACTCTG	112 bp
		Fw	CTGGATTTGTAGTTCCGTGGG T	
NM_003856.4	*IL1RL1*(s) (h)	Fw	GCTCCGTCACTGACTCCA AG	108 bp
		Rv	ACACATGAGGCAGTTGGTGA	
NM_002609.4	*PDGFRB* (h)	Fw	CGCAAAGAAAGTGGGCGGCT	101 bp
		Rv	TGCAGGATGGAGCGGATGTGGT	

### Immunocytochemistry and high-throughput image analysis

At endpoint cells were fixed using 4% paraformaldehyde solution. After washing in phosphate buffered saline (PBS) with 0.2% Triton X-100™ (Sigma) (PBS-T) plates were incubated with primary antibodies diluted in immunobuffer (1% goat or donkey serum (Gibco), 0.2% Triton X-100™, and 0.04% thiomersal (Sigma) in PBS) overnight at 4°C. Dilutions of primary antibodies are listed in Supplementary Table 1. Cells were washed again in PBS-T and incubated with fluorescently conjugated secondary antibodies (1:500; A-21206**,** R37119, A-11055 (Thermofisher)) 2–3 h at room temperature then rinsed. Nuclei were detected using Hoechst 33258 (bisBenzamide H, Sigma-Aldrich) 1:10,000 with secondary antibodies. Quantitative analysis of intensity measures and scoring of positively stained cells was performed using the Cell Scoring, Multiwavelength Cell Scoring, and Nuclear Translocation modules on MetaXpress® software (Molecular Devices) as previously described [71].

### ELISA

Conditioned media was collected at endpoint and centrifuged at 160*xg* for five minutes to collect possible cells and debris. Supernatant was obtained and stored at -20°C. Concentrations of soluble ST2 were determined using the Human ST2/IL-33 R DuoSet ELISA (R&D Systems) as per manufacturer’s instructions.

### Cytokine screen

Pericytes were plated into 12-well plates at 50,000 cells per mL, until 90% confluent (2–3 days). After a half media change of complete DMEM, cells were treated with cytokines at 10 × the concentrations in Table 2 for 24 h. At endpoint cells were rinsed in PBS and RNA extracted as described above for RT-qPCR.

**Table 2:** Cytokines, growth factors and other compounds used to treat pericytes in the current study. Abbreviations: LPS, lipopolysaccharide; IL1β, interleukin 1 beta; IL4, interleukin 4; IL6, interleukin 6; IL8, interleukin 8; IL17, interleukin 17; IL33, interleukin 33; IFNα, interferon alpha, IFNγ, interferon gamma; IGF1, insulin like growth factor 1; IGF2, insulin like growth factor 2; PDGF-AA, platelet-derived growth factor AA; PDGF-BB, platelet-derived growth factor BB; TNFα, tumor necrosis factor a; TGFα, transforming growth factor alpha; TGFβ1, transforming growth factor beta 1; VEGFA, vascular endothelial growth factor A; VEGFB, vascular endothelial growth factor B

Treatment	Concentration	Catalogue #	Source
LPS	10 ng/mL	L6143	Sigma-Aldrich
IL1β	10 ng/mL	200-01B	PeproTech
IL4	10 ng/mL	200–04	PeproTech
IL6	10 ng/mL	200–06	PeproTech
IL8	10 ng/mL	200–08	PeproTech
IL17	10 ng/mL	200–17	PeproTech
IL33	10 ng/mL	200–33	PeproTech
IFNα	10 ng/mL	106-12Y	Primegene
IFNγ	10 ng/mL	300–02	PeproTech
IGF1	10 ng/mL	100–11	PeproTech
IGF2	10 ng/mL	100–12	PeproTech
PDGF-AA	10 ng/mL	100-13A	PeproTech
PDGF-BB	10 ng/mL	100-14B	PeproTech
TNFα	10 ng/mL	300-01A	PeproTech
TGFα	10 ng/mL	100-16A	PeproTech
TGFβ1	10 ng/mL	100-21C	PeproTech
VEGFA	10 ng/mL	100–20	PeproTech
VEGFb	10 ng/mL	105-16Y	Primegene

### Animal models

EAE model: All experiments were performed under Institutional Animal Care and Use Committee approval at University of California San Francisco (UCSF) and University of California San Diego (UCSD), and mice were housed in a 12-h light/dark cycle with 2–5 mice per cage.

EAE was induced in C57BL/6 female mice by injecting the MOG35-55 peptide in an emulsion from Hooke Laboratories. Briefly, on the first day, mice were injected subcutaneously with the MOG35-55 peptide mixed with complete Freud’s adjuvant (EK-2110) and, simultaneously, 100 μL pertussis toxin was injected intraperitoneally. On the next day, the pertussis toxin injection was repeated. Control mice did not receive injections. Mice were then scored for EAE symptoms daily. Mice were anesthetized through an intraperitoneal injection of a ketamine/xylene cocktail and then perfused transcardially with PBS followed by 4% paraformaldehyde (Electron Microscopy Sciences 15,714-S) using a Dynamax peristaltic pump. Brains and spinal cords were fixed in formalin for 24 h and then stored in 70% EtOH until being embedded in paraffin and sectioned at 10 μm.

### Photothrombotic stroke

Stroke mouse brains were obtained from 10–12-week-old C57BL/6J male mice from the Hercus Taieri Research Unit, University of Otago, Dunedin, New Zealand. Mice were housed in groups of 3–5 mice under conditions of 12-h light/dark cycle (light off at 6 a.m.) with free access to water and food. All experiments were approved by the University of Otago Animal Ethics Committee and carried out in accordance with the NIH Animal Protection Guidelines for the care and use of animals for scientific purposes.

Focal stroke was induced in the mice as previously reported [72]. All mice were given a dose of Temgesic at the beginning of the surgery. Under isoflurane anesthesia (2–2.5% in O_2_) mice (*n* = 25 total, 5 per timepoint (1, 3, 5, 7 days post stroke and 5 sham controls) were placed in a stereotactic apparatus. A cold light source (KL1500 LCD, Zeiss) attached to a 40 × objective giving a 2-mm diameter illumination was positioned 1.5 mm lateral from Bregma on top of the exposed intact skull. Photothrombosis was induced by administration of 0.2 mL Rose Bengal solution (10 g/L in normal saline, intraperitoneally (Sigma)(*n* = 5 per timepoint post stroke) and 0.2 mL saline for sham animals (*n* = 5) which was allowed to circulate for 5 min. The brain was then illuminated for 15 min, while maintaining body temperature at 37.0 ± 0.3 °C by a heating pad (Harvard apparatus). The mice were returned to their home cage after a short recovery period and held under normal housing conditions for 1, 3, 5 or 7 days post-stroke. Based on all prior studies in our laboratory, we have 95–97% success / survival rate in young mice and 90–92% success/survival rate in aged mice. For the present study, no mice were excluded from these studies.

### Dual in situ hybridization and immunofluorescence

Both in situ hybridization with RNAscope probes and immunofluorescence were carried out as previously described [73, 74]. Briefly, the RNAscope 2.5 HD Detection kit (Red) (ACD Bio cat# 322,350) was used per manufacturer’s instructions for custom made probes against *IL1RL1* (NM_010743.3, encoding mouse *IL1RL1*(s), cat# ADV446301, and NM_001025602.3, encoding mouse *IL1RL1* (m), cat# ADV446291) or *PDGFb* (NM_011057.3, cat# 424651), positive control probe *Ppib* (cat# 313911), and negative control probe dapB (cat# 310043)(ACD Bio). Fast red chromogen was added to sections before washing in MilliQ water and blocking in 10% normal donkey serum (NDS) (Gibco cat# 16,210,072). Probes are designed to be specific to transcript targets as reviewed [75, 76], and images of both positive and negative probes are included in the supplementary data (Supplementary Fig. 1). Antibodies for either PDGFRβ (R&D Biosystems cat# AF385), CD13 (R&D Biosystems cat# AF2335), or IL-33 (R&D Biosystems, cat# AF3626) diluted in 1% NDS were then added overnight at 4 °C. Secondary antibody incubation (AlexaFluor647 cat# A32849, 1:500 diluted in 1% NDS) and Lectin (Vector laboratories, cat# DL-1174–1, 1:100) for 3 h at room temperature was followed by incubation with Hoechst 33,342 (Invitrogen cat# H3570 at 1:20,000) for 5 min at room temperature. Slides were coverslipped with ProLong Diamond antifade reagent (Invitrogen cat# P36965) and stored at 4 °C prior to imaging.

### Imaging and analysis

Following RNAscope in situ hybridization and immunofluorescence staining, sections were imaged with the Zeiss Axio Imager Z2 running MetaSystems Metafer5 Vslide software with a 10 × objective (0.45 numerical aperture). The RNAscope puncta were captured with both the green transmitted light (GTL) filter and the Texas Red (TxRed), whilst cell nuclei, lectin (FITC) and PDGFRβ(Cy5)-labelled cells were captured under their respective fluorescence filters. For quantification of RNAscope signal within specific cell types in EAE sections, we adopted a custom ImageJ module as previously described [73]. Pericyte-specific expression of *IL1RL1*(m) and *IL1RL1*(s) was quantified by detecting puncta within masked PDGFRβ staining before normalising to total PDGFRβ-positive area as performed previously [11].

### Statistical analysis

GraphPad Prism was used to assess statistical significance in the current study. Datasets were tested for normality with the Shapiro–Wilk test, followed by ANOVA, or Two-way ANOVA and correction for multiple comparisons as indicated in the figure legends.

## Supplementary Information

Additional file1 (PDF 1837 kb)

## Data Availability

The publicly available RNAseq dataset analysed here is available from the Gene Expression Omnibus ( [GSE189712](https:/www.ncbi.nlm.nih.gov/geo/query/acc.cgi?acc=GSE189712)). All data presented in this manuscript can be made available by the corresponding author upon reasonable request.
