# Supplementary material for: Central nervous system pericytes express soluble ST2 in inflammation and injury
Source: Mol Brain. 2026 Mar 19;19:31. doi: 10.1186/s13041-026-01291-5 (PMC13123163; doi:10.1186/s13041-026-01291-5)
Supplement: Supplementary file 1 — Additional file1 (PDF 1837 kb) [file 13041_2026_1291_MOESM1_ESM.pdf]

Supplementary Data

Supplementary Table 1

**Target**	**Catalogue #**	**Company**	**Dilution**
pAkt	CS9271	Cell Signaling Technologies	1:200
c-jun	SC1694	Santa Cruz	1:500
EGR-1	CS4153	Cell Signaling Technologies	1:1000
pERK	CS4370	Cell Signaling Technologies	1:500
NFκB	SC8008	Santa Cruz Biotechnology	1:500
PDGFRβ	AF385	R & D Systems	1:1000

Antibodies used for immunocytochemistry in this study.

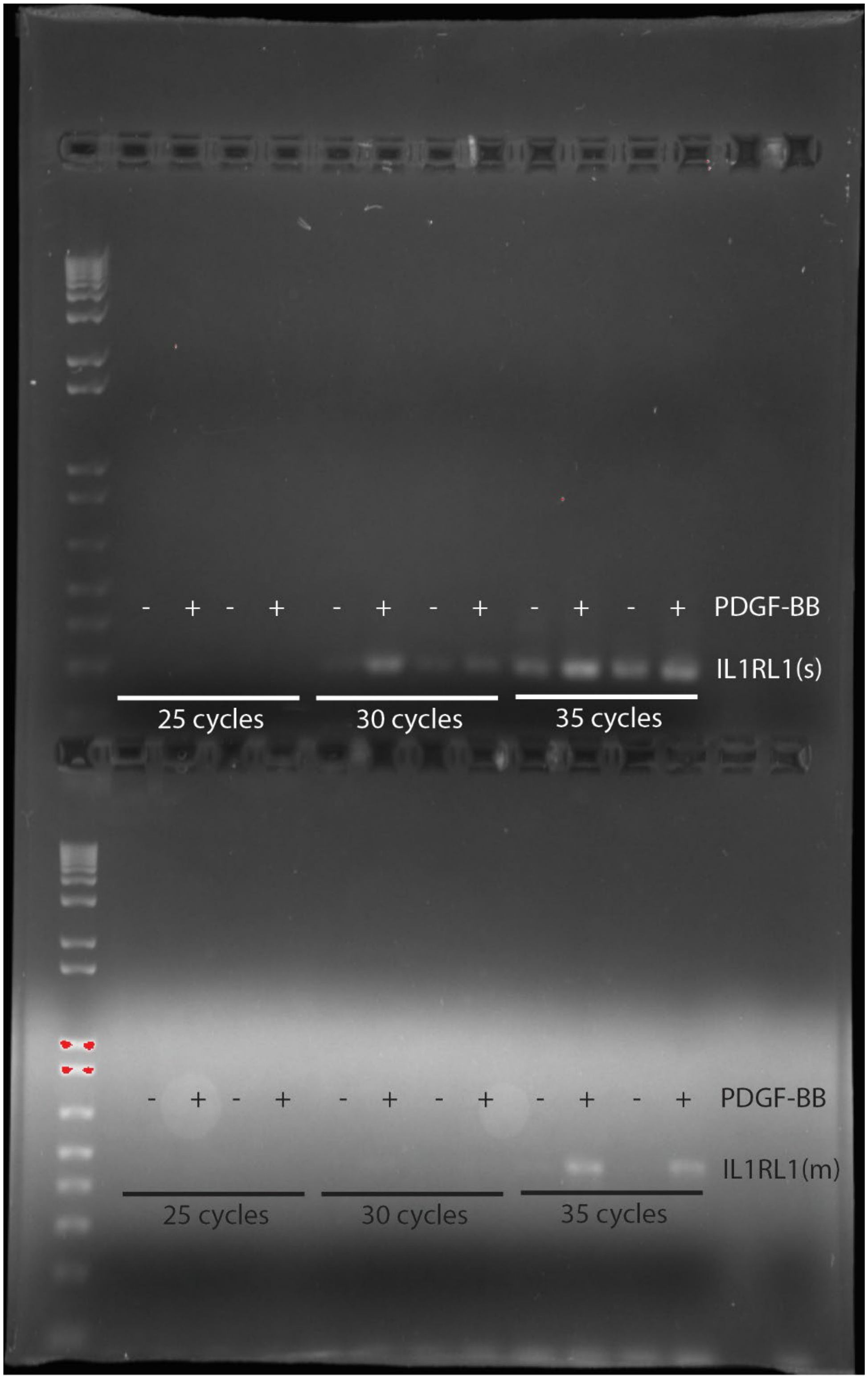

Supplementary Figure 1: Pericyte were treated with PDGF-BB (10ng/mL) for 24 hrs then RNA was extracted. Transcript levels of either soluble IL1RL1(s) or membrane IL1RL1(m) forms were measured with different primers for PCR.

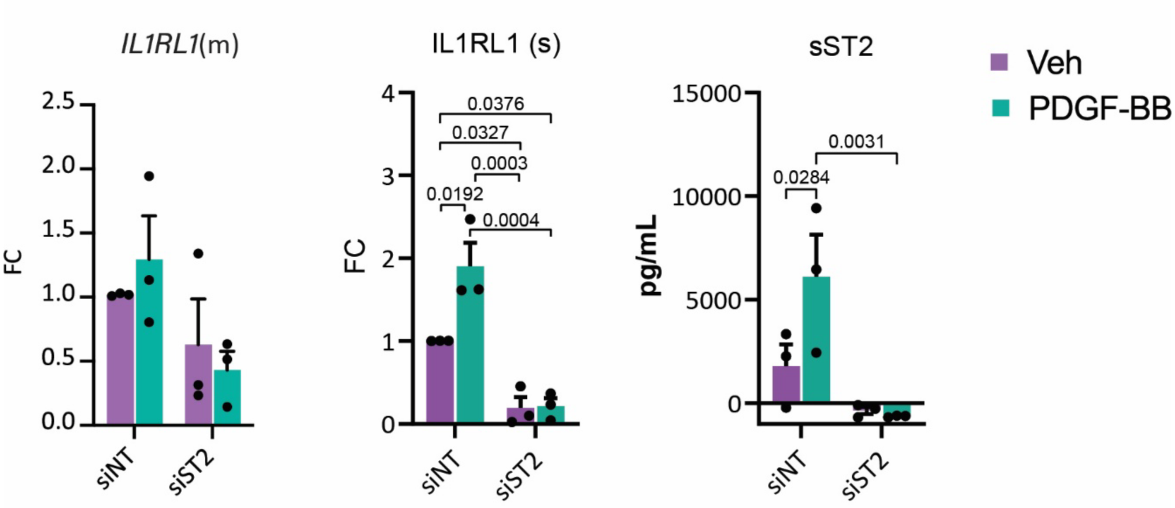

Supplementary Figure 2: Pericytes were treated with siRNA for either scrambled control or siST2, and gene expression of *IL1RL1*(m), or *IL1RL1*(s)was quantified with RT-qPCR, or protein levels of sST2 were quantified with ELISA. Statistical analysis was conducted with two-way ANOVA with Tukey's correction for multiple comparisons, (*$p<0.05$, **$p<0.01$, ***$p<0.001$).

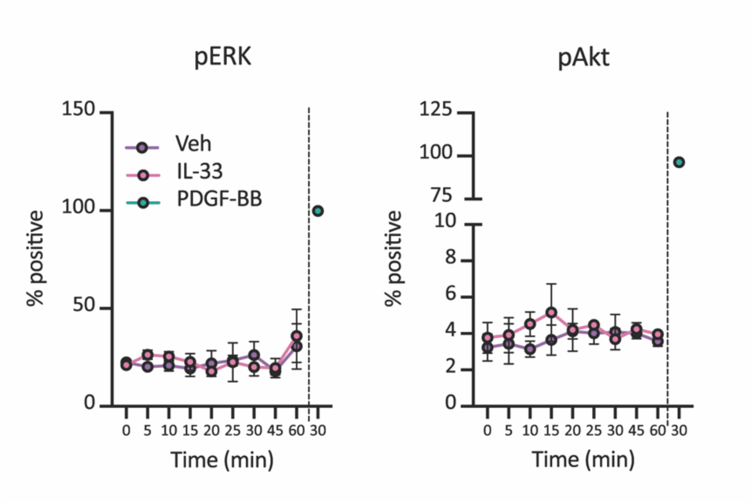

Supplementary Figure 3: Pericytes were treated with IL-33 or PDGF-BB (10 ng/mL) for the indicated times, fixed, and pERK and pAkt were quantified using immunocytochemistry (n=3 cases). Statistical analysis was conducted with two-way ANOVA with uncorrected Fisher's LSD.

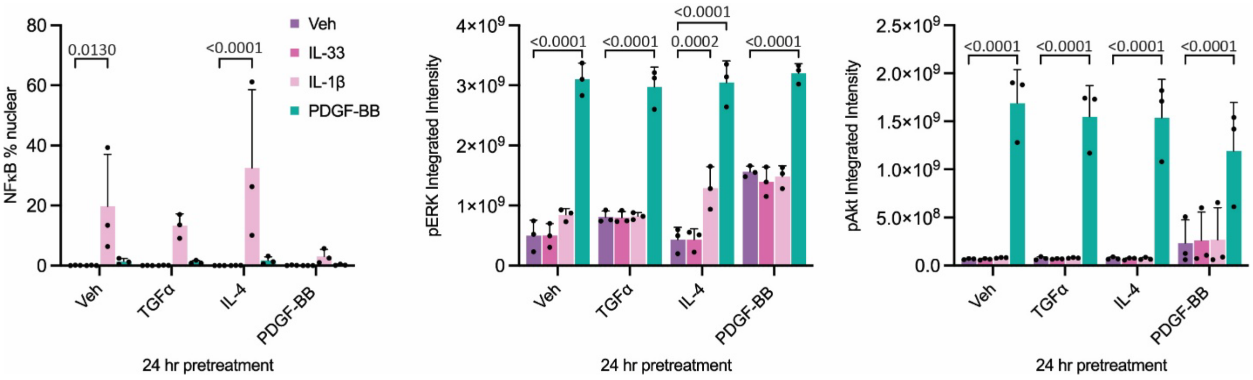

Supplementary Figure 4: Pericytes were treated with vehicle, TGFα, IL4 or PDGF-BB (all 10 ng/mL) for 24 hours. Cells were then treated with vehicle, IL33, IL1β or PDGF-BB (10 ng/mL) for 30 minutes (to assess pERK/pAkt) or 1 hour to measure NFκB nuclear translocation. Statistical analysis was conducted with two-way ANOVA with Dunnett's correction for multiple comparisons, (**p*<0.05, ***p*<0.01, ****p*<0.001).

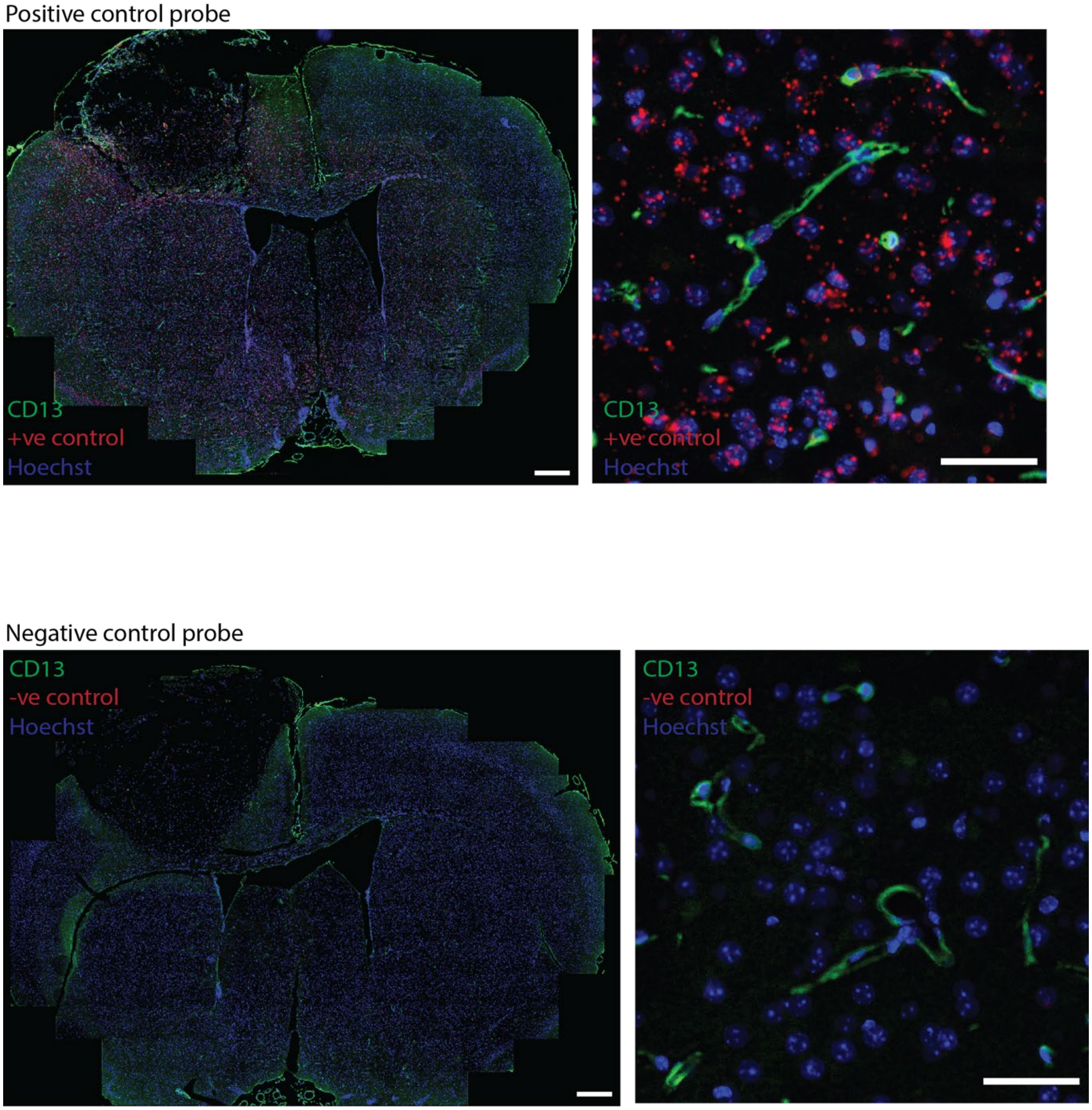

Supplementary Figure 5: Low magnification and high magnification images of CD13 staining and RNAscope positive and negative probes. Scale bar for left images (500 μm), and for right images (50 μm).

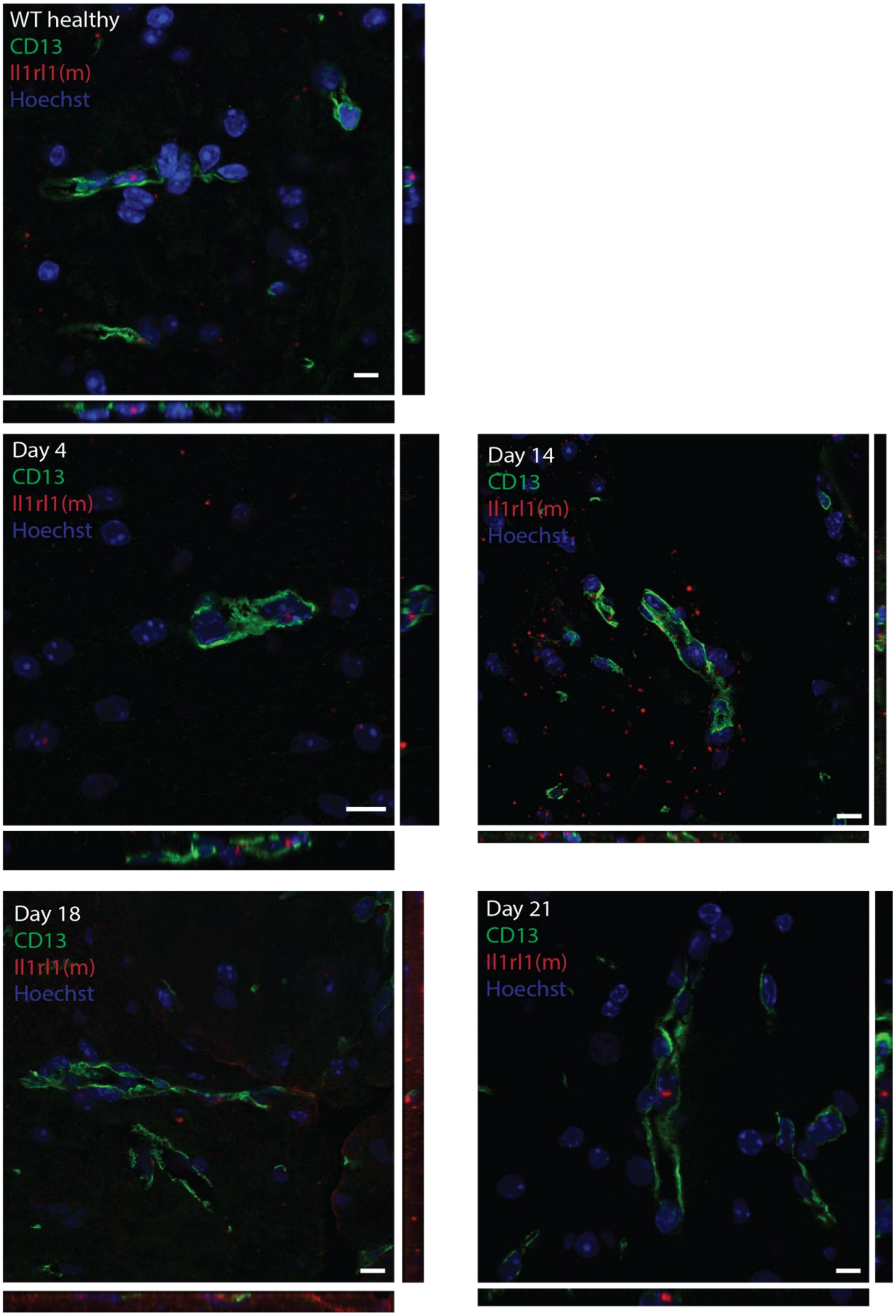

Supplementary Figure 6: Representative confocal images showing overlay of RNAscope probe *IL1RL1*(m) for the membrane-bound isoform of ST2 with pericyte marker CD13 in mouse spinal cord sections from the EAE model. Scale bars=10 µm.

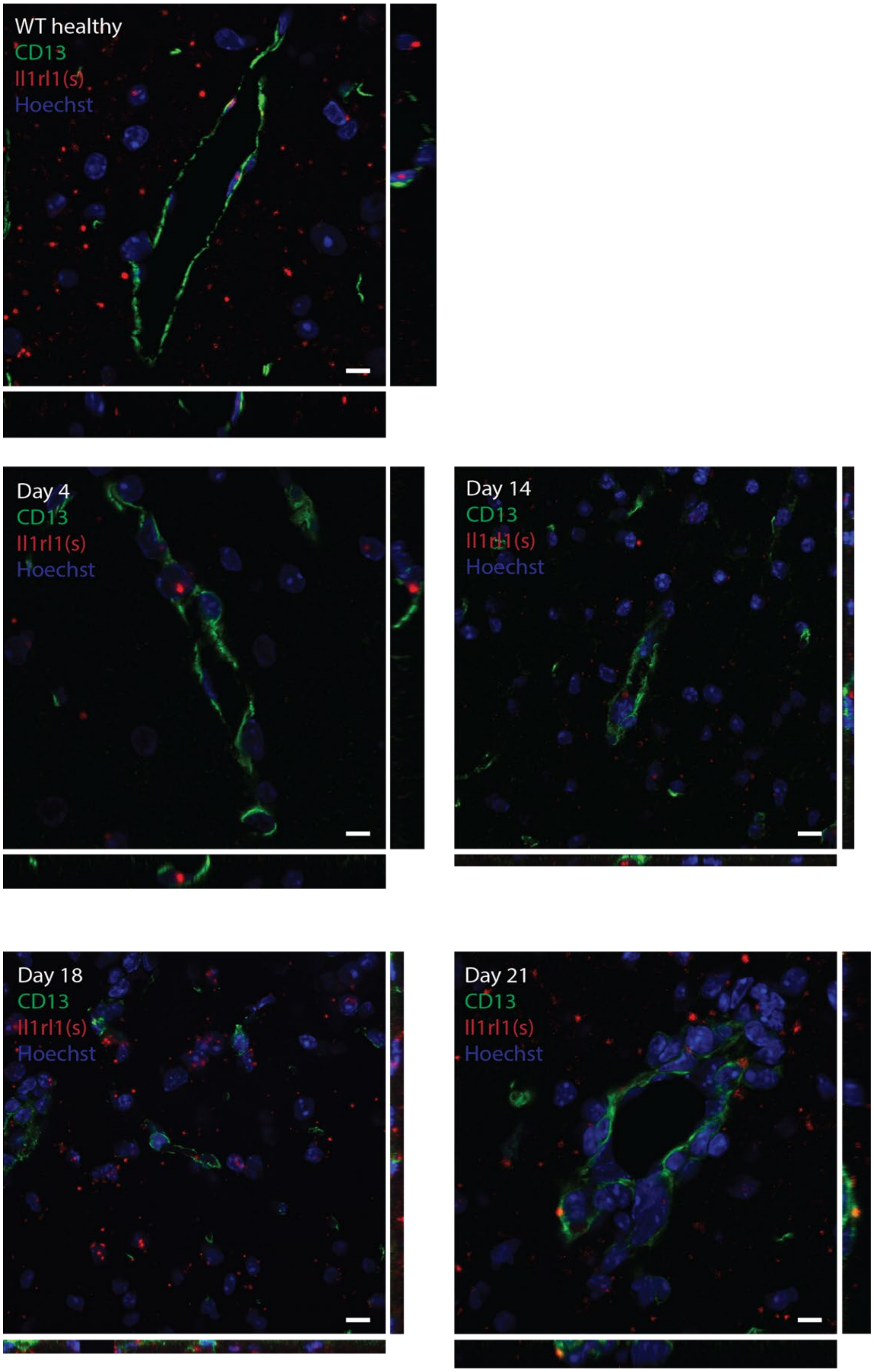

Supplementary Figure 7: Representative confocal images showing overlay of RNAscope probe *IL1RL1*(s) for the soluble isoform of ST2 with pericyte marker CD13 in mouse spinal cord sections from the EAE model. Scale bars= 10 μm.

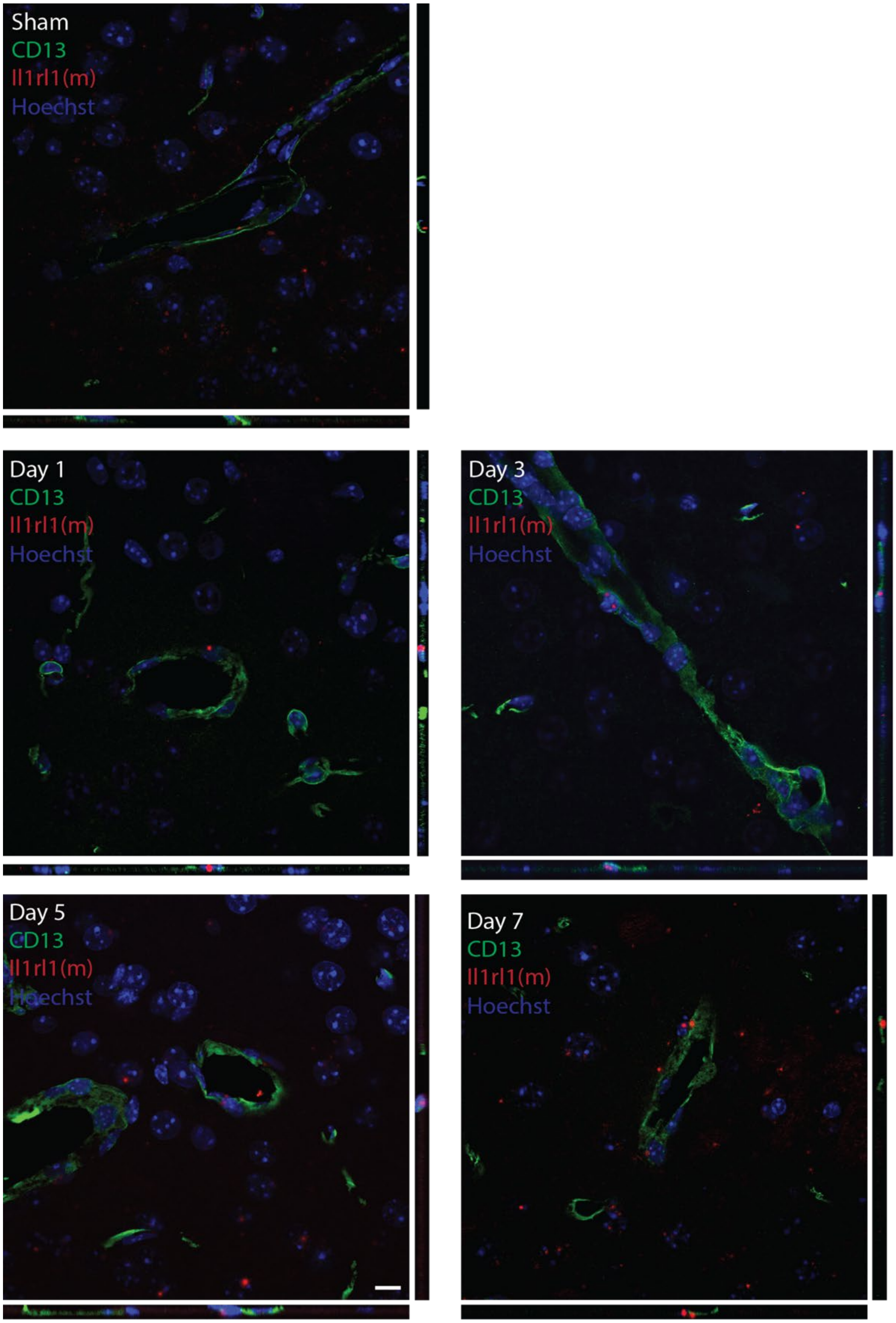

Supplementary Figure 8: Representative confocal images showing overlay of RNAscope probe *IL1RL1*(m), for the membrane-bound isoform of ST2 with pericyte marker CD13 in mouse cortex (outside the infarct area) sections from the stroke model. Scale bars=10 μm.

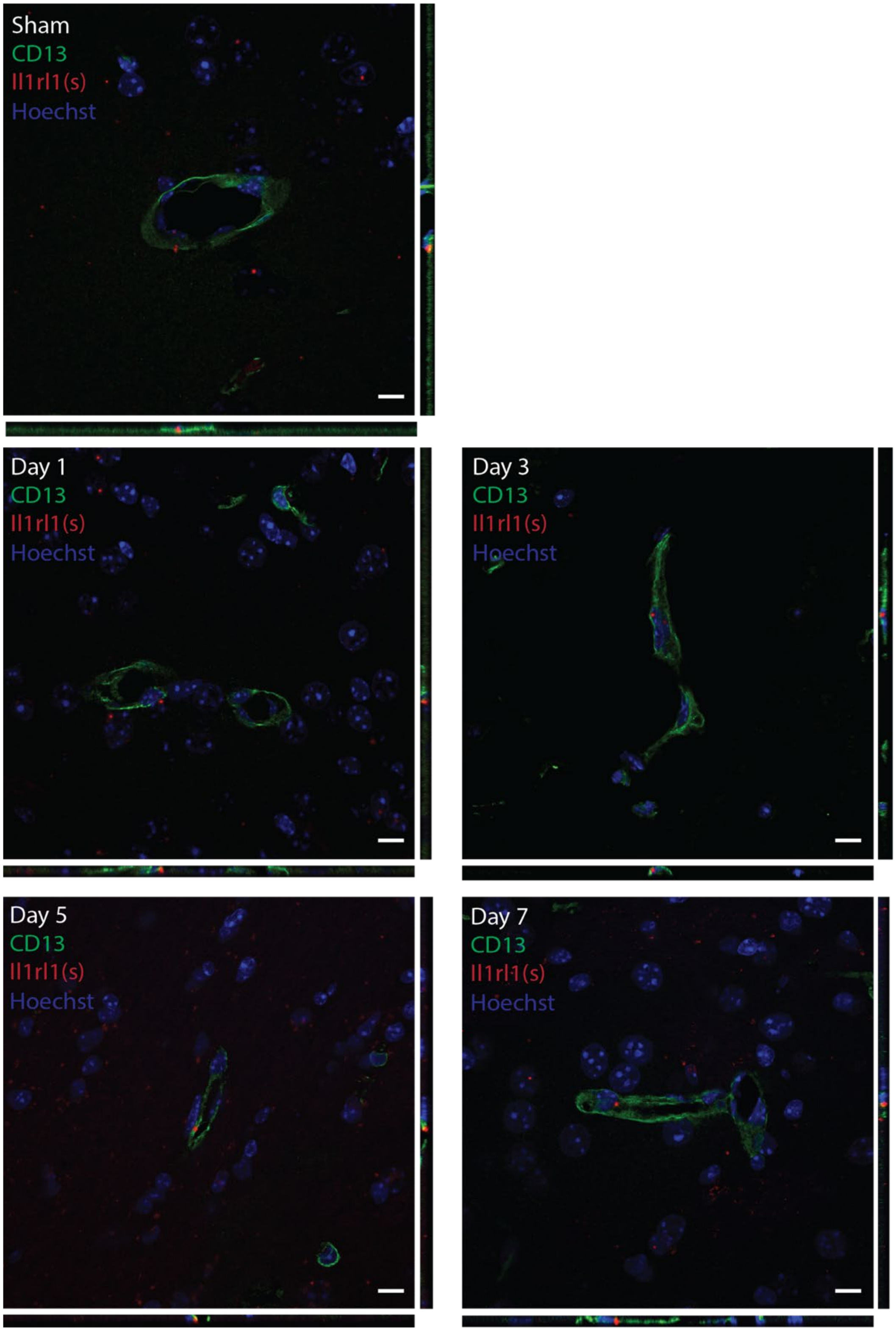

Supplementary Figure 9: Representative confocal images showing overlay of RNAscope probe *IL1RL1*(s) for the soluble isoform of ST2 with pericyte marker CD13, in mouse cortex (outside the infarct area) sections from the stroke model. Scale bars=10 μm.

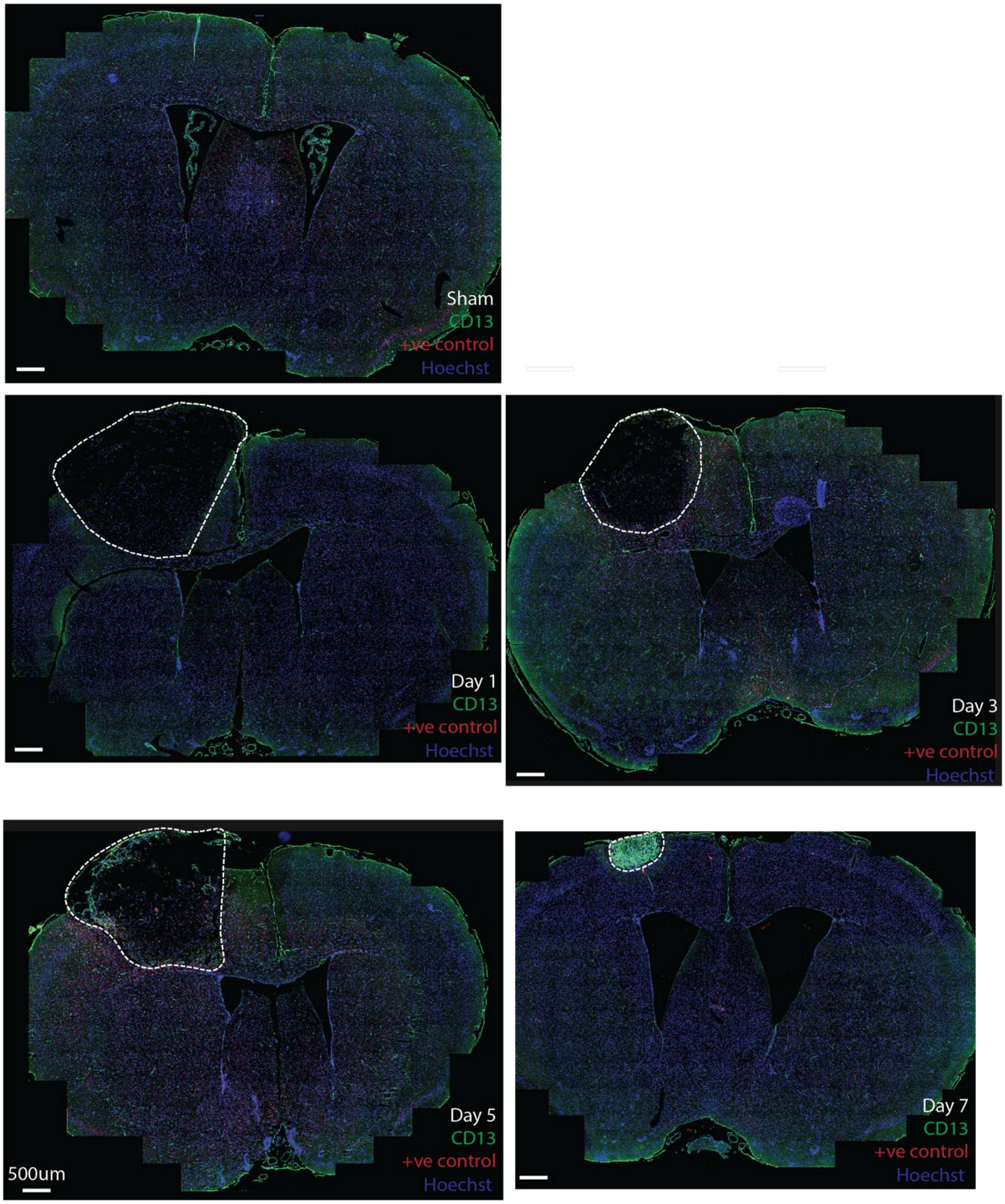

Supplementary Figure 10: Representative low magnification images showing overlay of RNAscope positive control probe co-labelled with pericyte marker CD13 in mouse cortex sections from the stroke model.
